# Resveratrol ameliorates maternal and post-weaning high-fat diet-induced nonalcoholic fatty liver disease via renin-angiotensin system

**DOI:** 10.1186/s12944-018-0824-3

**Published:** 2018-07-28

**Authors:** Mao-Meng Tiao, Yu-Ju Lin, Hong-Ren Yu, Jiunn-Ming Sheen, I-Chun Lin, Yun-Ju Lai, You-Lin Tain, Li-Tung Huang, Ching-Chou Tsai

**Affiliations:** 1 Department of Pediatrics, Kaohsiung Chang Gung Memorial Hospital, Chang Gung University, College of Medicine, Kaohsiung, 83301 Taiwan; 2 Department of Obstetrics and Gynecology, Kaohsiung Chang Gung Memorial Hospital, Chang Gung University, College of Medicine, 123 Ta-Pei Road, Niao Sung, Kaohsiung, 83301 Taiwan, Republic of China

**Keywords:** Nonalcoholic fatty liver disease (NAFLD), High-fat diet, Resveratrol, Renin-angiotensin system (RAS)

## Abstract

**Background:**

Nonalcoholic fatty liver disease (NAFLD) can develop in prenatal stages and can be exacerbated by exposure to a postnatal high-fat (HF) diet. We investigated the protective effects of resveratrol on prenatal and postnatal HF diet-induced NAFLD.

**Methods:**

Male Sprague–Dawley rat offspring were placed in five experimental groups (*n* = 10–12 per group): normal diet (VNF), maternal HF diet (ONF), postnatal HF diet (VHF), and maternal HF diet/postnatal HF diet (OHF). A therapeutic group with resveratrol for maternal HF diet/postnatal HF diet (OHFR) was used for comparison. Resveratrol (50 mg/kg/day) was dissolved in drinking water for offspring from post-weaning to postnatal day (PND) 120.

**Results:**

We found that HF/HF-induced NAFLD was prevented in adult offspring by the administration of resveratrol. Resveratrol administration mediated a protective effect on rats on HF/HF by regulating lipid metabolism, reducing oxidative stress and apoptosis, restoring nutrient-sensing pathways by increasing Sirt1 and leptin expression, and mediating the renin-angiotensin system (RAS) to decrease angiotensinogen, renin, ACE1, and AT1R levels and increased ACE2, AT2R and MAS1 levels compared to those in the OHF group.

**Conclusion:**

Our results suggest that a maternal and post-weaning HF diet increases liver steatosis and apoptosis via the RAS. Resveratrol might serve as a therapeutic target by mediating protective actions against NAFLD in offspring exposed to a combination of maternal and postnatal HF diet.

## Background

Epidemiological data support the hypothesis that metabolic syndrome is an independent risk factor for nonalcoholic fatty liver disease (NAFLD) [1, 2]. Recent studies also support the view that NAFLD may be a precursor of incident type 2 diabetes mellitus (T2DM) and metabolic syndrome [3, 4]. Fatty liver accumulation, inflammation, and insulin resistance are risk factors in the development of NAFLD [5]. NAFLD is defined by the presence of steatosis in > 5% of hepatocytes according to histological analysis or by a proton density fat fraction > 5.6% assessed using proton magnetic resonance spectroscopy or quantitative fat/water selective magnetic resonance imaging. NAFLD includes two pathologically distinct conditions with different prognoses: nonalcoholic fatty liver and non-alcoholic steatohepatitis; the latter covers a wide spectrum of disease severities, including fibrosis, cirrhosis, and hepatocellular carcinoma [6] Liver steatosis is a chronic liver disease that is common in NAFLD patients, characterized by a spectrum of hepatic pathologies that can lead to cirrhosis [7]. Our previous studies have shown that maternal or post-weaning high fat (HF) diets lead to a variety of chronic diseases in adult offspring, including liver steatosis [8, 9].

In recent years, evidence suggests that sirtuins play important roles in regulating fatty liver disease-related metabolic processes [10]. The renin-angiotensin system (RAS) is recognized as an important modulator of body metabolic processes. Angiotensin II (Ang II), the biologically active component of RAS, acts through two receptor subtypes, the angiotensin type 1 receptor (AT1) and angiotensin type 2 receptor (AT2). The discovery of angiotensin-converting enzyme 2 (ACE2) has a potential therapeutic role in RAS modulation. The importance of the balance between ACE/Ang-II/AT1 and ACE2/Ang-(1–7)/Mas is in avoiding metabolic disease of the liver [11]. The multiple insults act together on genetically predisposed subjects to induce NAFLD. Such insults include insulin resistance, hormones secreted from the adipose tissue, nutritional factors, gut microbiota, and genetic and epigenetic factors. It results from a complex interaction between a specific genetic background and multiple environmental/metabolic hits [12–14]. A previous study reported that antioxidants (specifically resveratrol) ameliorated fibrosis and inflammation in a mouse model of nonalcoholic steatohepatitis [15]. In humans, some studies found resveratrol supplements may benefit patients with NAFLD [16–18]. However, other studies reported resveratrol administration did not significantly improve NAFLD compared with placebo [19–21].

The purpose of this study was to investigate the effects of a maternal high-fat diet during pregnancy, as well as in combination with a postnatal high-fat diet, on the metabolic profiles of adult male offspring. The protective effect of resveratrol against NAFLD was also assessed.

## Methods

### Animals

This animal study was carried out following the Guide for the Care and Use of Laboratory Animals by the National Institutes of Health. The protocol was approved by the Institutional Animal Care and Use Committee of the Kaohsiung Chang Gung Memorial Hospital, Kaohsiung, Taiwan. Sprague–Dawley (SD) rats (12–16 weeks old; BioLASCO Taiwan Co, Ltd., Taipei, Taiwan) were housed in the animal care facility in Chang Gung Memorial Hospital. They were housed under a 12 h light/dark cycle with lights on at 7 a.m. and with temperature maintained at 22 °C. Pregnant rats were checked for litters every 10 h. Sprague–Dawley female rats were allowed to mate with male rats for 24 h. One day later, female rats were separated from the male rats and housed individually in a standard cage. Female rats were weight-matched and assigned to receive either a normal diet or high-fat diet (58% high-fat diet, Research Diet, New Brunswick, NJ, USA, Country, D12331) ad libitum for 5 weeks before mating, during gestation, and during lactation. Male offspring were weaned at 5 weeks of age and put onto either the normal diet or high-fat diet from weaning to 4 months of age.

### Animal grouping

Five experimental groups were considered: prenatal normal diet/postnatal normal-fat diet (VNF) as a control group, maternal high-fat diet/postnatal normal-fat diet (ONF), maternal normal diet/postnatal high-fat diet (VHF), maternal obesity high-fat diet/postnatal high-fat diet (OHF), and therapeutic group on a maternal high-fat /postnatal high-fat and resveratrol diet (OHFR). The control group (VNF) consisted of pregnant SD rats on a normal diet before pregnancy until lactation. Offspring were weaned onto a normal diet until PND 120. The ONF group consisted of pregnant SD rats on a high-fat diet 7–8 weeks before mating until lactation. Offspring were weaned on a normal diet until PND 120. The VHF group consisted of pregnant SD rats fed a normal diet before pregnancy until lactation. Offspring were weaned on a high-fat diet until PND 120. The OHF group consisted of pregnant SD rats on a high-fat diet 7–8 weeks before mating until lactation. Offspring were weaned on a high-fat diet until PND 120. The OHFR group consisted of pregnant SD rats on a high-fat for 7–8 weeks before mating until lactation. Offspring were weaned on a high-fat diet until PND 120. Resveratrol (50 mg/kg/day) was dissolved in the drinking water for offspring from post-weaning to postnatal day (PND) 120.

### Tissue collection and blood sampling

Male offspring were weighed and sacrificed at PND 120 (*n* = 10–12 per group) and plasma and liver tissue samples were collected. Enzyme-linked immunosorbent assay (ELISA) was carried out on the plasma samples for the detection of triglyceride, aspartate transaminase, alanine aminotransferase, adiponectin, cholesterol, and HDL, according to the manufacturer’s instructions. Leptin concentrations were determined by the ELISA assay kit (Biovendor, RD291001200R, Karasek, Brno, Czech Republic).

### Hematoxylin and eosin staining

Livers were dissected and fixed in 4% paraformaldehyde at 4 °C overnight. The fixed tissues were dehydrated in a gradient of ethanol (70, 75, 85, 90, 95, and 100%), hyalinized in xylene, and embedded in paraffin wax at 55 °C. Sections were cut at 3 μm and stained with an H&E Staining Kit (ScyTek Laboratories, West Logan, USA). A Leica DMI-3000 microscope equipped with a digital camera was used to observe the histologic lesions.

### TUNEL assay

Tissues were immersed in 4% paraformaldehyde in 0.1 mol/L phosphate buffer and fixed overnight at 4 °C. Fixed tissues were paraffin-embedded, cut into 3 μm thick transverse sections, and mounted on slides. An apoptosis detection kit (Roche, 11,684,817,910, Mannheim, Germany) was used according to the manufacturer’s instructions. Sections were visualized with 3, 3-diaminobenzidine tetrahydrochloride and counterstained with Gill’s hematoxylin. Positive cells and total hepatocytes were counted from 20 randomly selected high-power fields (200×) from each section under light microscopy and the positive rates of TUNEL were calculated. A total of 500 hepatocytes from each rat were used to count positively stained cells.

### Hepatic triglyceride assay

Liver tissues (350–400 mg) were homogenized and centrifuged at 10,000×*g* for 10 min at 4 °C. The supernatant was assayed using a triglyceride by triglyceride colorimetric assay kit (Cayman, 1,001,303, Ann Arbor, Michigan, USA), according to the manufacturer’s instructions.

### Reverse transcription and real-time PCR

Total RNA was extracted from 100 mg frozen liver samples using Trizol reagent (Invitrogen; Boston, MA, USA). RNA was quantified by A260 and its integrity verified by agarose gel electrophoresis using ethidium bromide for visualization. For reverse transcription, 1 μg of total RNA and Oligo (dT) primers were heated at 65 °C for 5 min and then cooled on ice. This mixture was combined with 500 μmol/L of each of dATP, dTTP, dCTP, and dGTP, 10 mmol/L DTT, 40 units of RNaseOUT recombinant ribonuclease inhibitor (Invitrogen, 10,777,019, Boston, MA, USA), and 100 units SuperScript III reverse transcriptase (Invitrogen, 18,080,093, Boston, MA, USA). Aliquots were taken for immediate use in PCR and the remainder of cDNA was stored at − 20 °C. RNA expression was measured by real-time PCR in a Lightcycler 480 (Roche Diagnostics; Mannheim, Germany). Amplification was carried out using specific primer pairs listed in the Table 1. GAPDH was used as the internal control to normalize the relative amount of cDNA in each reaction. The PCR reaction was performed using the following cycling protocol: 95 °C for 5 min, followed by 45 cycles of 95 °C for 5 s, 60 °C for 15 s, and 72 °C for 20 s. Dissociation curves were run after amplification to identify specific PCR products. RNA expression levels were normalized to GAPDH RNA levels and calculated according to the ΔΔCt method.

**Table 1:** Oligonucleotide sequence 5' → 3'

Gene of interest	Forward	Reverse
SIRT1	5' TGGAGCAGGTTGCAGGAATCCA	5' TGGCTTCATGATGGCAAGTGGC
AT1R	5' ACCAGGTCAAGTGGATTTCG	5' ATCACCACCAAGCTGTTTCC
AT2R	5' CAATCTGGCTGTGGCTGACTT	5' TGCACATCACAGGTCCAAAGA
ACE1	5' CACCGGCAAGGTCTGCTT	5' CTTGGCATAGTTTCGTGAGGAA
ACE2	5' GCCAGGAGATGACCGGAAA	5' CTGAAGTCTCCATGTCCCAGATC
MAS	5' CATCTCTCCTCTCGGCTTTGTG	5' CCTCATCCGGAAGCAAAGG
Renin	5' AACATTACCAGGGCAACTTTCACT	5' ACCCCCTTCATGGTGATCTG
Angiotensionogen	5' GTGTGATGCCTCCTGTGTA	5' TGCTGCTCATCATTTATTCTCAGTTA
Leptin receptor	5' ACTGGGACATAGAGTGCTGG	5' GTTGCACTGGACAGTCTGAA
GAPDH	5' TAAAGAACAGGCTCTTAGCACA	5' AGTCTTGGAAATGGATTGTCTC

### Western blot

Livers were dissected and subsequently frozen in liquid nitrogen. The tissue of each liver was homogenized in a buffer and centrifuged at 14,000×g. Protein (65 μg) from the supernatant of each sample was separated by SDS-PAGE and transferred onto polyvinylidene difluoride (PVDF) membranes. Membranes were blocked in TBST buffer containing 10% non-fat milk for 1 h at room temperature. Immunoblotting was performed by incubating the blocked membrane overnight at 4 °C with a monoclonal mouse leptin receptor antibody (Gene Tex/GTX37636, Irvine, CA, USA), Sirt1 antibody (cell signaling /#9475, Danver, MA, USA), ACE2 antibody (abcam/ab108252, Cambridge, MA, USA), and malondialdehyde (MDA) antibody (abcam/ab27642, Cambridge, MA, USA). The membranes were then incubated with secondary HRP conjugated anti-rabbit antibody (1:5000; Jackson Immuno Research, West Grove, PA USA*)* or anti-mouse antibody (1:10,000; Jackson Immuno Research, West Grove, PA USA) for 1 h at room temperature. Western blots were visualized using an ECL kit (Perlcin Elmer In./NEL 105001EA, Boston, MA, USA).

### Statistical analysis

SPSS 15.0 for Windows was used for statistical analysis. For most parameters, statistical analysis was carried out using analysis of variance (ANOVA) with a Bonferroni post hoc test. The data presented are the mean ± SE. The level of statistical significance was set at *P* < 0.05.

## Results

### Body weight and biochemistry parameters of rats increased in the OHF group and decreased in the OHFR group

As shown in Table 2, body weight and liver weight were significantly higher in the VHF and OHF group than those in the VNF group (*P* < 0.05). These effects were reversed in the OHFR group after resveratrol treatment (*P* < 0.05). The liver/body ratio showed no significant difference between OHF and the other groups, except for the VNF group.

**Table 2:** Weight and biochemical parameters of the animal subjects in their corresponding experimental groups

Weight (gm)	395.88 ± 3.98 ^#,†,◎^	398.67 ± 6.18 ^#,†,◎^	631.53 ± 17.27^*,†,◎^	714.64 ± 11.96^*,#,◎^	558.89 ± 18.65^*,#,†^
Liver weight (gm)	11.28 ± 0.23 ^#,†,◎^	11.20 ± 0.30 ^#,†,◎^	17.78 ± 0.52^*,◎^	18.92 ± 0.62^*,◎^	15.01 ± 0.46^*,#,†^
Liver/body (%)	2.85 ± 0.04 ^†^	2.81 ± 0.05	2.82 ± 0.06	2.64 ± 0.06^*^	2.72 ± 0.10
AST (U/L)	112.92 ± 6.56 ^#,†,◎^	98.28 ± 6.36 ^#,†,◎^	257.70 ± 24.88^*,◎^	232.29 ± 22.87^*^	200.21 ± 18.97^*,#^
ALT (U/L)	32.92 ± 1.04 ^#,†,◎^	33.67 ± 1.69 ^#,†,◎^	153.57 ± 18.72^*,◎^	113.86 ± 13.91^*^	110.50 ± 15.57^*,#^
T-Cholesterol (mg/dL)	47.56 ± 2.19 ^#^	51.67 ± 2.82 ^†^	57.48 ± 2.18^*^	64.078 ± 3.43^*,◎^	50.22 ± 2.43 ^†^
Triglyceride (mg/dL)	83.76 ± 6.08	91.61 ± 10.02^◎^	75.26 ± 8.78 ^†^	101.79 ± 12.72^◎^	64.86 ± 4.83 ^†^
HDL (mg/dL)	31.28 ± 2.27 ^#,†^	32.72 ± 2.18 ^#,†^	39.30 ± 1.83^*,◎^	42.93 ± 2.40^*,◎^	32.00 ± 1.89 ^#,†^

*AST* aspartate transaminase, *ALT* alanine aminotransferase, *VNF* prenatal/postnatal normal diet group, *ONF* prenatal high fat diet/postnatal normal diet group, *VHF* prenatal normal diet/postnatal high fat diet group, *OHF* prenatal/postnatal high-fat diet group, *OHFR* prenatal/postnatal high-fat diet/resveratrol group

*P < 0.05 compared to the VNF group, #*P* < 0.05 compared to the VHF group, ^†^*P* < 0.05 compared to the OHF group, ^◎^*P* < 0.05 compared to the OHFR group

Animals in the OHF group showed higher AST and ALT levels than in the VNF or ONF groups. These were not significantly reversed after resveratrol administration. Furthermore, cholesterol levels were significantly higher in the OHF group than in the ONF group, and the triglyceride levels were higher than in the VHF group. These effects were reversed after resveratrol administration (Table 2).

### Maternal and post-weaning HF diet caused a leptin increase in the liver that was improved by resveratrol treatment

**Fig. 1:**
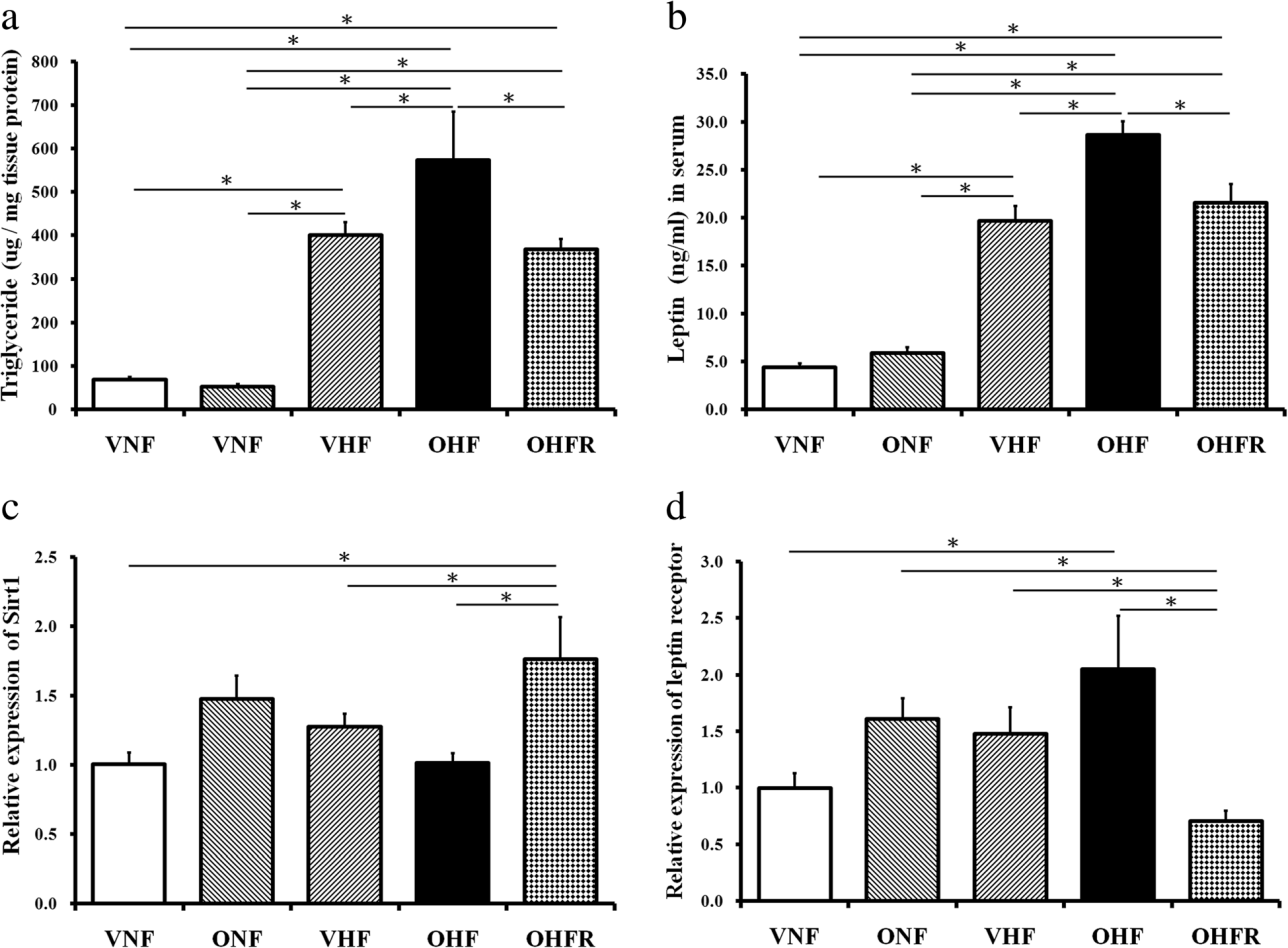
Resveratrol administration improved maternal and post weaning HF diet-induced fatty acid metabolism. **a** Triglyceride expression in liver tissue. **b** Leptin levels in serum. **c** Liver mRNA levels of leptin receptor. **d** Liver mRNA expression level of Sirt-1 than that of the OHF group. *P < 0.05. VNF: normal diet, ONF: maternal high-fat diet, VHF: postnatal high-fat diet, OHF: maternal high-fat diet/postnatal high-fat diet, and OHFR: OHF with resveratrol

Triglyceride expression in liver tissue was significantly increased in the OHF group and decreased in the OHFR group (Fig. 1a). The OHF group also showed the highest leptin levels, which were significantly reduced in the OHFR group than in the OHF and VNF groups (*P* < 0.05) (Fig. 1b). The liver mRNA level of the leptin receptor increased by a maternal and post-weaning HF diet, which resveratrol was found to prevent (*P* < 0.05) (Fig. 1c). The liver mRNA level of Sirt1 was reduced by a maternal and post-weaning HF diet. Resveratrol therapy significantly restored and increased the levels of Sirt1 (*P* < 0.05) (Fig. 1d).

### Reduced lipid accumulation in hepatic cells after resveratrol therapy

**Fig. 2:**
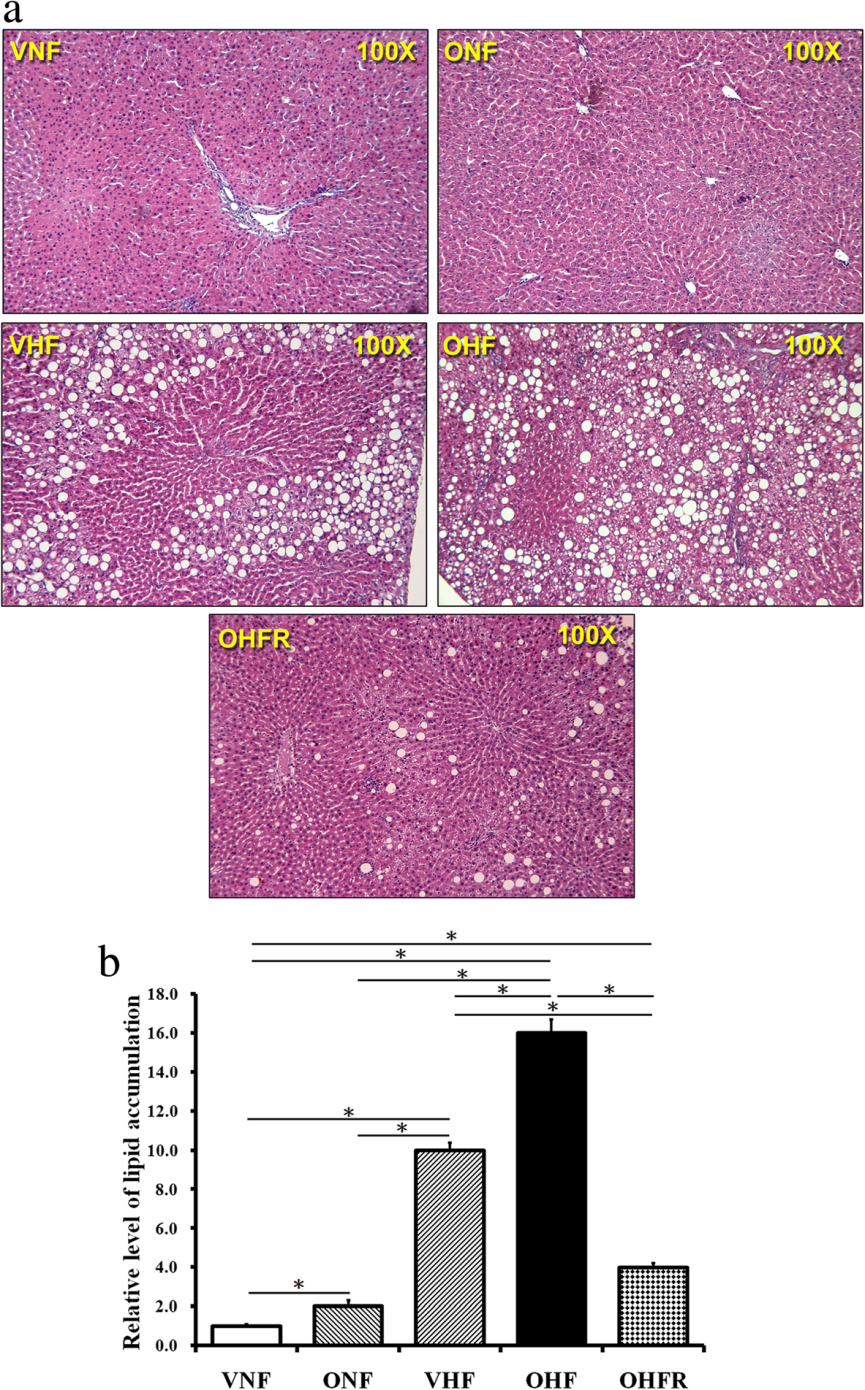
Investigation of hepatic lipid accumulation by HE staining and quantification. Lipid accumulates in hepatocytes as vacuoles. **a** Light micrographs showing immunostaining with HE staining in hepatic cells. **b** Quantitative analysis of lipid accumulation in liver samples per microscopic field (100×). Resveratrol therapy led to a lower level of lipid droplets in the OHFR group than in the OHF group. *P < 0.05. VNF: normal diet, ONF: maternal high-fat diet, VHF: postnatal high-fat diet, OHF: maternal high-fat diet/postnatal high-fat diet, and OHFR: OHF with resveratrol

Lipid accumulates in hepatocytes as vacuoles. H&E staining of liver tissues (Fig. 2a) showed a greater number of lipid droplets in the OHF group than in the other four groups, indicating a synergistic effect between a maternal and post-weaning HF diet. In addition, the results showed that resveratrol therapy reduced the level of lipid droplets in the OHFR group than in the OHF group (Fig. 2b). This suggested that resveratrol therapy was efficient in reducing liver lipid storage by attenuating liver steatosis in rats on a maternal and post-weaning HF diet.

### Decreasing apoptosis after resveratrol therapy

**Fig. 3:**
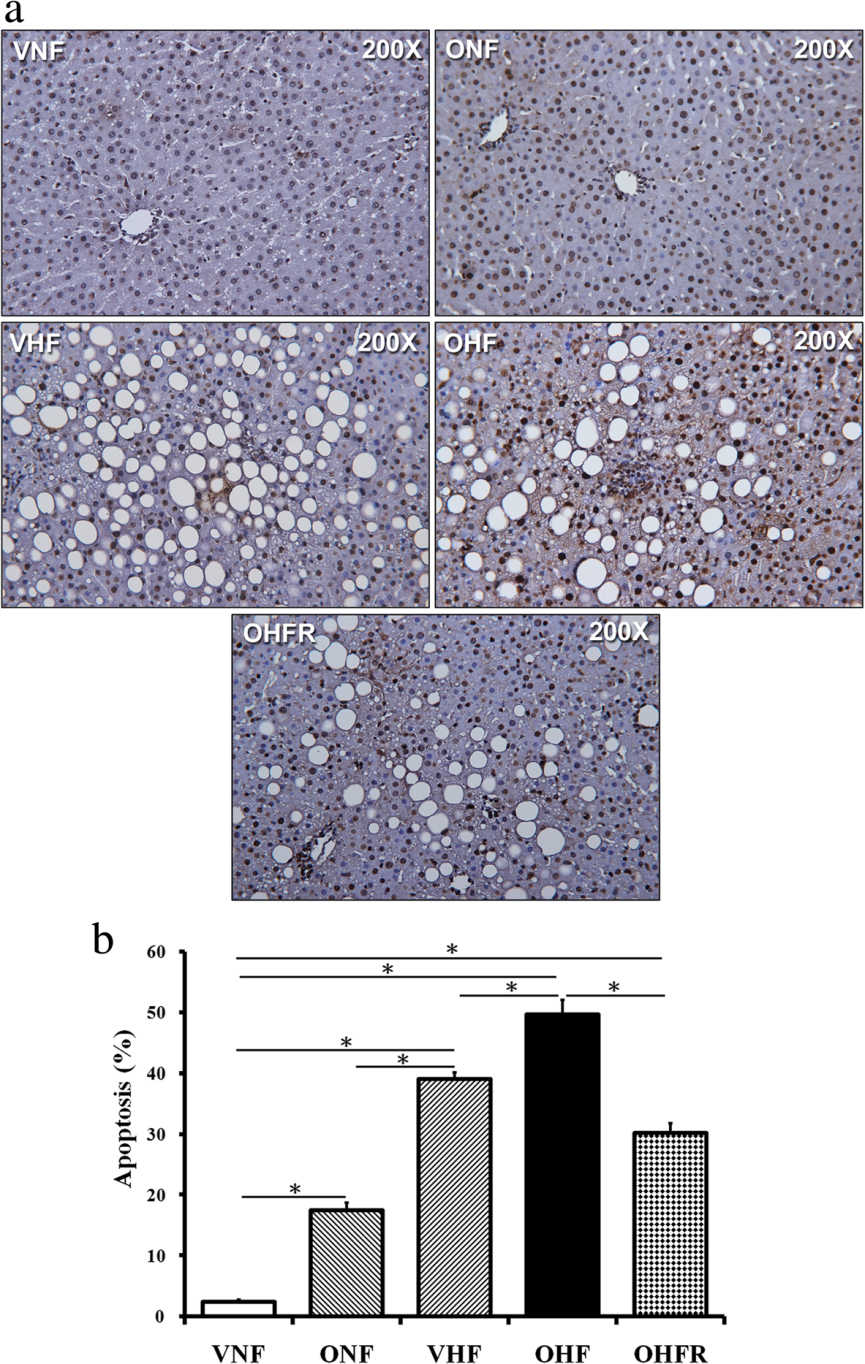
TUNEL assay to analyze the role of apoptosis in liver damage. **a** TUNEL staining was stronger in the OHF, VHF, and ONF groups than in the vehicle-treated group, while the OHFR group presented reduced TUNEL staining in comparison with the OHF group (magnification, × 200; bar = 30 μm). **b** Semi-quantitative analysis of TUNEL stained cells. *P < 0.05. VNF: normal diet, ONF: maternal high-fat diet, VHF: postnatal high-fat diet, OHF: maternal high-fat diet/postnatal high-fat diet, and OHFR: OHF with resveratrol

Activation of apoptotic pathways was based on the extent of TUNEL staining (Fig. 3a). TUNEL staining revealed a significantly greater proportion of apoptotic cells in the OHF group than in the other four groups. Following resveratrol therapy in the OHFR group, the degree of TUNEL staining was decreased in comparison with that in the OHF group (Fig. 3b). These findings suggest that resveratrol therapy is efficient in reducing liver cell apoptosis in rats with a maternal and post weaning HF diet.

### Renin-angiotensin system modulation in rats with maternal and post weaning HF diet and resveratrol therapy

**Fig. 4:**
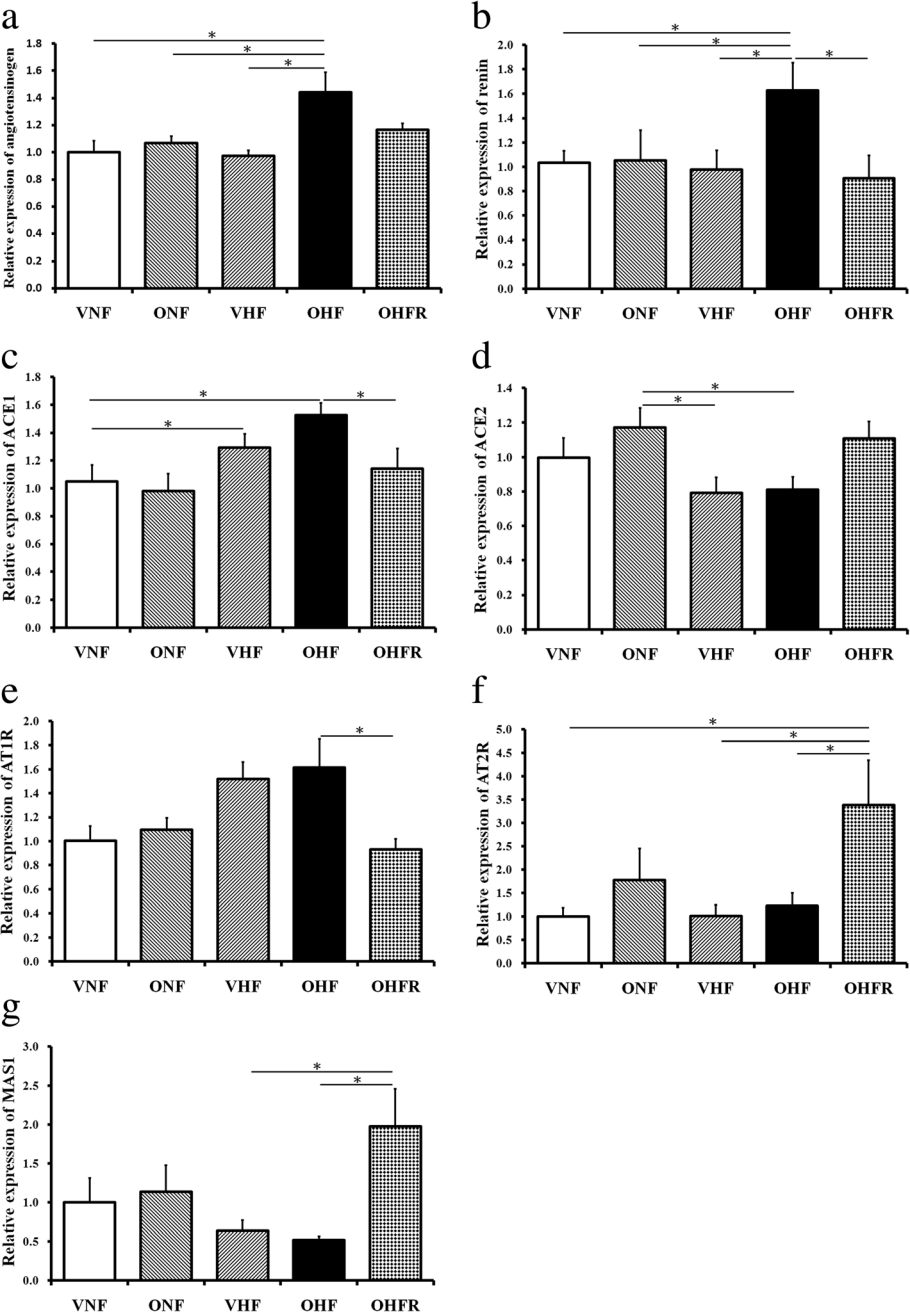
Effect of maternal and post weaning HF diet and resveratrol on mRNA expression of RAS. Liver mRNA levels of (**a**) angiotensinogen, (**b**) renin, (**c**) ACE1, and (**e**) AT1R significantly decreased in the OHFR group compared to those in the OHF group. Liver mRNA levels of (**d**) ACE2, (**f**) AT2R, and (**g**) MAS1 significantly increased in the OHFR group compared to those in the OHF group. *P < 0.05. VNF: normal diet, ONF: maternal high-fat diet, VHF: postnatal high-fat diet, OHF: maternal high-fat diet/postnatal high-fat diet, and OHFR:OHF with resveratrol

In addition to the apoptosis pathway, the deleterious effects on the liver of the ACE/Ang II/AT1 axis are well documented in the literature [11, 22]. Our study showed mRNA levels of angiotensinogen, renin, ACE1, and AT1R in the liver were increased by a maternal and post weaning HF diet in the OHF group. However, resveratrol therapy was found to significantly decrease the level of angiotensinogen, renin, ACE1, and AT1R in the OHFR group compared to that in the OHF group (Fig. 4a–d). mRNA levels of ACE2, AT2R, and MAS1 in the liver were significantly decreased in the OHF group, and resveratrol therapy was found to increase these levels in the OHFR group (Fig. 4e–g). These findings suggest that resveratrol therapy interacts with liver cells in rats with a maternal and post weaning HF diet via renin-angiotensin system modulation.

### Protein expression levels of leptin receptor, Sirt1, ACE2, and MDA in maternal and post weaning HF diet and resveratrol therapy

**Fig. 5:**
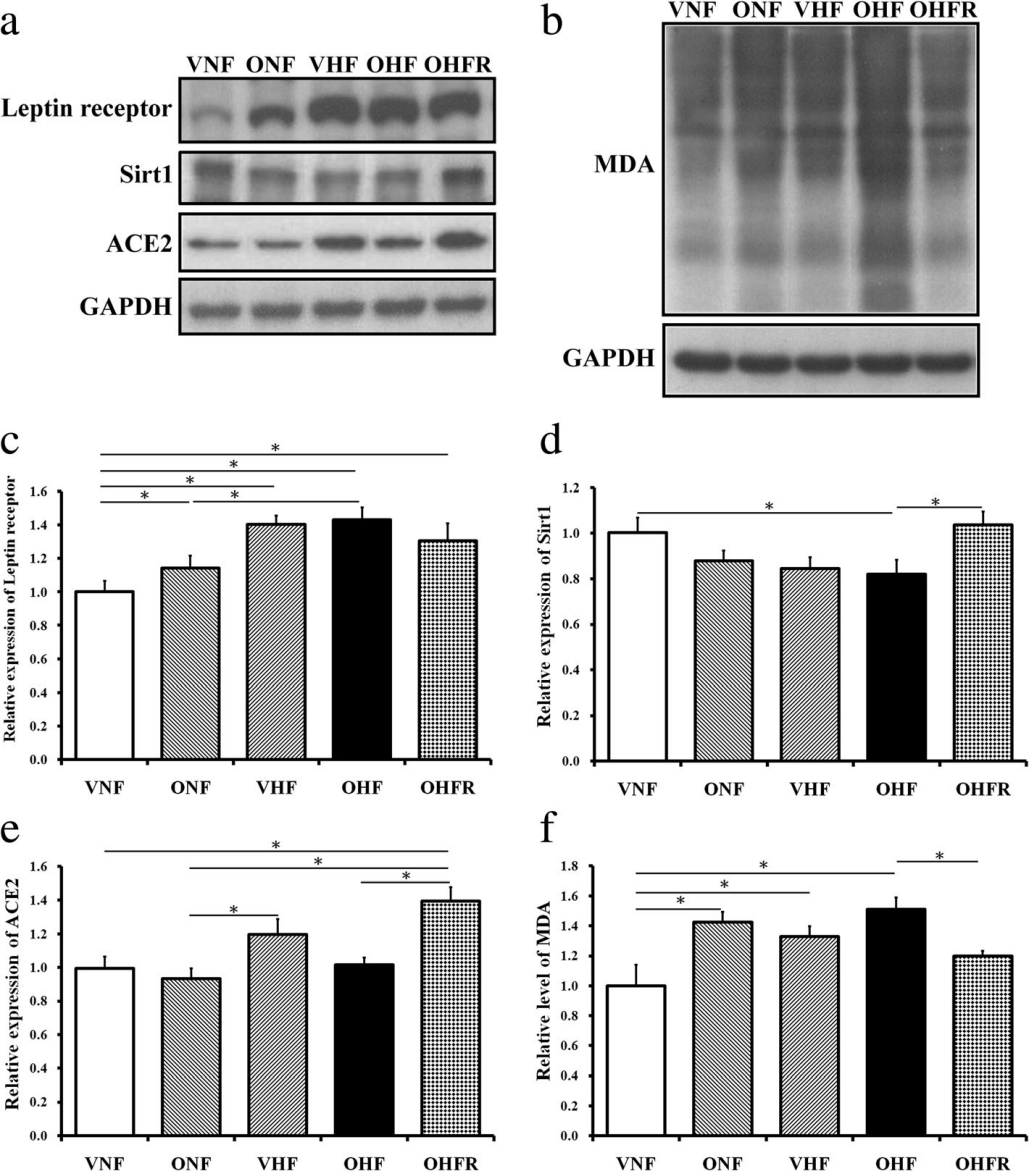
Western blotting shows changes in protein expression between the five groups. (**a**, **c**) Protein expression of the leptin receptor and (**b**, **d**) MDA protein expression. **d** Sirt 1 protein expression, and (**e**) ACE2 protein expression. *P < 0.05. VNF: normal diet. ONF: maternal high-fat diet. VHF: postnatal high-fat diet, OHF: maternal high-fat diet/postnatal high-fat diet, and OHFR: OHF with resveratrol

MDA results from the lipid peroxidation of polyunsaturated fatty acids and is used as a biomarker to measure the levels of oxidative stress [23]. Among the other four groups, the OHF group showed the highest protein expression for the leptin receptor and MDA (Fig. 5). The OHFR group showed a decrease in the leptin receptor and MDA protein expression (Fig. 5a-c,f). In contrast, ACE2 and Sirt1 protein expression in the OHFR group was at the highest level among the other four groups. The OHF group showed decreased Sirt1 protein expression. Resveratrol therapy was found to increase Sirt 1 and ACE2 protein expression (*P* < 0.05, Fig. 5d, e).

## Discussion

Our results suggest that resveratrol therapy led to a decrease in the levels of cholesterol and triglyceride in the OHF group. These results are similar to the observations of our previous study [24]. This study demonstrated that resveratrol was efficient in treating fatty liver disease induced by a maternal high-fat diet during pregnancy in combination with a postnatal high-fat diet. Resveratrol administration was found to: i) reduce liver lipid storage; ii) decrease expression levels of MDA, leptin, and leptin receptor in the liver; iii) decrease apoptosis in the liver, possibly by the regulation of RAS; iv) decrease the expression of angiotensinogen, renin, ACE1, and AT1R in the liver; v) increase the expression of ACE2, AT2R, and MAS1 in the liver; and vi) increase Sirt1 protein expression in the liver.

### Leptin resistance is not involved in resveratrol administration

Insulin and leptin resistance are known to play a role in metabolic syndromes and nonalcoholic fatty liver disease (NAFLD) [24]. Leptin resistance has previously been reported in conjunction with high triglyceride levels and low leptin receptor levels [25]. This was found in the OHF group, which showed higher plasma triglyceride and leptin levels (Fig. 1a, b) and higher leptin receptor mRNA expression in the liver than the other four groups (Fig. 1d). With respect to the OHF group, resveratrol administration reduced serum triglyceride and leptin levels, as well as leptin receptor mRNA expression in rats on a maternal and post weaning HF diet (*P* < 0.05, Fig. 1a, b, d). The OHF group was found to have increased levels of the leptin and leptin receptor, which is not related to the usual description of leptin resistance.

### Changes in oxidative stress and apoptosis with resveratrol administration

Oxidative stress is another major contributor to disease progression in NAFLD [26], and previous studies have indicated that hepatic MDA levels increase in high-fat diet-induced NAFLD [27, 28]. In the present study, it was demonstrated that resveratrol administration reduced oxidative stress by lowering MDA levels in rats on a maternal and post weaning HF diet. Apoptosis is the main process contributing to disease progression in NAFLD [29] and our previous study revealed increased liver apoptosis in rats with prenatal dexamethasone exposure that were receiving a high-fat diet postnatally [8]. In the present study, TUNEL staining revealed a significantly greater proportion of apoptotic cells in the OHF group than in the other four groups. Following resveratrol therapy, the degree of TUNEL staining, indicative of liver cell apoptosis, was lesser than that of the OHF group. Resveratrol had a protective effect against liver cell apoptosis in this study.

### RAS in hepatic lipid metabolism via resveratrol administration

Ang-(1–7) can be formed directly from Ang I by neprilysin or from Ang II by prolyl oligopeptidase, prolyl-carboxypeptidase, or the newly discovered enzyme ACE2. Some researchers suggest that hepatic fibrosis is associated with RAS activation (increased Ang II/AT1) and that Ang-(1–7) plays a protective role against hepatic fibrosis [24]. Moreover, ACE2 negatively regulates the deleterious arm of the activated renin-angiotensin system by degrading Ang II to Ang-(1–7) [25]. It has been reported that RAS is related to hepatic steatosis. Angiotensin-converting enzyme 2/angiotensin-(1–7)/Mas axis activates Akt signaling to ameliorate hepatic steatosis [26]. Reactive oxygen species (ROS)-generating systems, such as nicotinamide adenine dinucleotide phosphate oxidase, can increase proinflammatory and profibrogenic gene expression as well as protein activity [27]. Ang II promotes the development and progression of NAFLD in the transgenic Ren2 rat model by increasing hepatic ROS [28]. Ang-(1–7) has been found to decrease liver gluconeogenesis [30], and the Mas receptor is an essential component of the insulin receptor signaling pathway [31].

Several studies support a counter-regulatory role for Ang-(1–7) by opposing the vascular and proliferative effects of Ang II [32, 33] and many other AT1 receptor-mediated actions [26]. RAS axis activation in cirrhotic human livers and in rat liver injury models are associated with the upregulation of ACE2 and Mas, which leads to increased circulating Ang-(1–7) levels, most likely as a protective response [34–37]. ACE2, together with Ang-(1–7) and the Mas receptor, acts as a counterbalance to RAS during metabolic liver injuries [33, 38]. A previous study reported that ACE2 deficiency promotes adipose tissue inflammation and augments obesity-induced glucose intolerance. Several studies that have focused on ACE2 highlight the importance of seeking therapies that increase angiotensin-converting enzyme activity in the prevention and treatment of diabetes and its complications [39]. The present study showed increased mRNA levels of angiotensinogen, renin, ACE1, and AT1R in the liver in the OHF group. Resveratrol therapy was found to significantly decrease the levels of angiotensinogen, renin, ACE1, and AT1R. On the other hand, resveratrol significantly reduced the mRNA levels of ACE2, AT2R, and MAS1 in the liver in the OHF group, but increased these levels in the OHFR group.

Our findings suggest that resveratrol therapy modulates the RAS to prevent the development of maternal and post weaning HF diet-induced NAFLD.

### Nutrient-sensing pathways in resveratrol administration

Lipid metabolism disorders are an important predisposing factor to fatty liver disease pathogenesis, which is characterized by excessive lipid accumulation in the liver. Among seven mammalian sirtuins, sirtuin 1 (Sirt1) is the most extensively studied and plays a role in both alcoholic and nonalcoholic fatty liver diseases. Sirt1 has a beneficial effect by regulating hepatic lipid metabolism, controlling hepatic oxidative stress, and hepatic inflammation through deacetylating transcriptional regulators against the progression of fatty liver disease [10]. Sirt1 also protects against a high-fat diet or alcohol consumption-induced hepatic steatosis via various metabolic pathways [10, 40–43]. Increasingly, evidence has demonstrated that Sirt1 acts as a key metabolic/energy sensor that directly couples the cellular metabolic/energy status (via an intracellular NAD+/NADH ratio) to regulate the transcriptional activity and/or gene expression of several crucial transcription factors and transcription co-activators that are involved in metabolic homeostasis [44–48]. In the present study, resveratrol administration was shown to reduce oxidative stress by increasing Sirt1 levels in rats with maternal and post weaning HF diets.

## Conclusions

Our study showed that a combination of maternal and post weaning HF diet increases liver steatosis and apoptosis. Resveratrol therapy resulted in several protective actions against fatty liver in offspring exposed to both a maternal and post weaning HF diet, including a regulated lipid metabolism, reduced oxidative stress, reduced apoptosis, restoration of nutrient-sensing pathways via increasing Sirt1, and modulation of renin-angiotensin system.

## Abbreviations

MDA: malondialdehyde
NAFLD: nonalcoholic fatty liver disease
TUNEL: TdT-mediated dUTP-biotin nick end-labeling
